# Supplementary material for: Unique Evolution of SARS-CoV-2 in the Second Large Cruise Ship Cluster in Japan
Source: Microorganisms. 2022 Jan 4;10(1):99. doi: 10.3390/microorganisms10010099 (PMC8778844; doi:10.3390/microorganisms10010099)
Supplement: Supplementary file 1 [file microorganisms-10-00099-s001.zip › microorganisms-1532731-supplementary.pdf]

Supplementary Materials

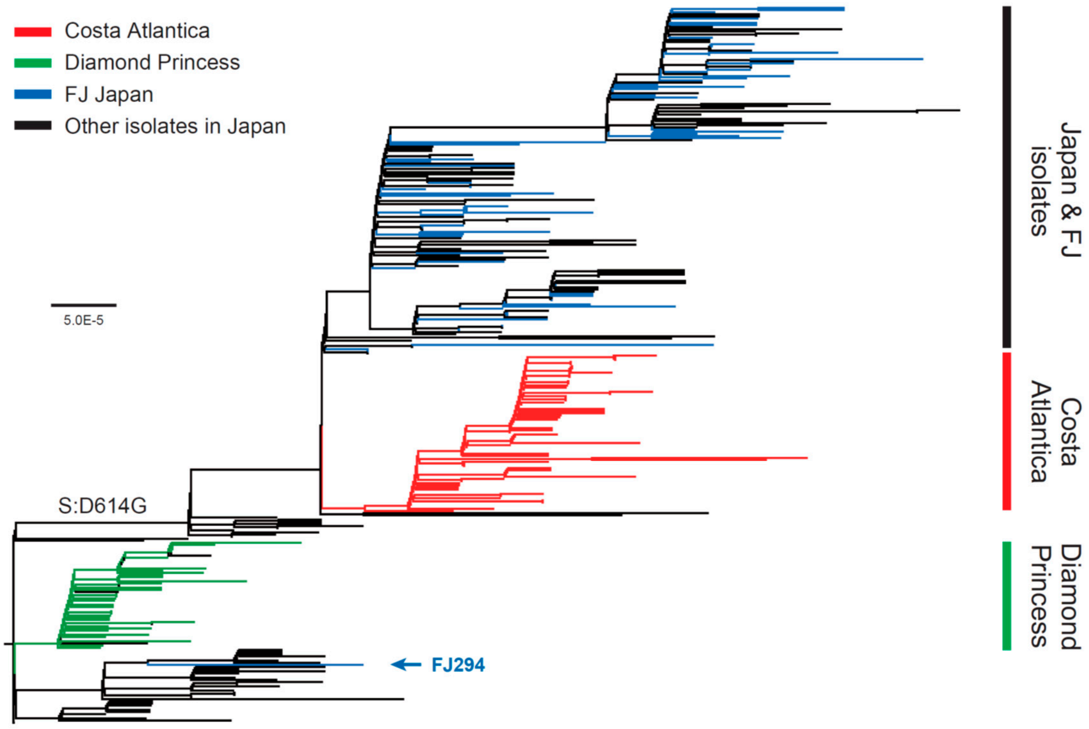

Figure S1. Maximum likelihood phylogeny of SARS-CoV-2 whole-genome sequences detected in the Costa Atlantica and Diamond Princess cruise ship clusters together with the reference sequences only from Japan. Scale bar depicts nucleotide substitutions per site.

Table S1. Diagnostic tests of crew members in Costa Atlantica in April, 2020.

**Costa Atlantica**				**Diamond Princess**			
Date	Tested	Positive	%	Date	Tested	Positive	%
Apr 20	4 [1]	1	25.0	Feb	3,618	712	19.7
Apr 21	57	33	57.9				
Apr 22	66	14	21.2				
Apr 23	208	43	20.7				
Apr 24	289 [2]	57	19.7				
Apr 25	1 [2]	0	0				
Total	623 [3]	148	23.8				

[1] Samples were tested by RT-qPCR.

[2] The number of test samples included re-test samples.

[3] Re-test samples were excluded.

Table S2. Acquired common amino acid substitutions between groups.

Group	Number	Mutation	Protein	Substitution
CA	1	T4346C	ORF1a NSP3	S543P
FJ	35			
DP	1	C9474T	ORF1a NSP4	A307V
FJ	1			
CA	1	G11083T	ORF1a NSP6	L37F
DP	71 [1]			
FJ	2			
CA	1	G15982T	ORF1b NSP12b	V839L
FJ	1			
DP	1	C21575T	S	L5F
FJ	2			

[1] The originally introduced strain into the DP cruise ship possessed this mutation.
FJ, FJ Japan; CA, Costa Atlantica; DP, Diamond Princess.

Table S3. Characteristics of the haplotype of each SARS-CoV-2 group.

Group	Haplotype	
	Segregating sites	Informative sites
FJ	154	36
CA	72	20
DP	48	5
England	211	94
California	641	241
France	564	281
Paris-LD	258	71
Milan-LD	296	79
Moscow-LD	195	44
Austria-SSE	34	1

FJ, FJ Japan; CA, Costa Atlantica; DP, Diamond Princess; LD, Lockdown; SSE, Superspreading event.
